# Conformational alteration in glycan induces phospholipase Cβ1 activation and angiogenesis

**DOI:** 10.1186/s12929-022-00889-w

**Published:** 2022-12-15

**Authors:** Sheng-Hung Wang, Jing-Yan Cheng, Hsiu-Hui Tsai, Tzu-Chi Lo, Jung-Tung Hung, Chun-Cheng Lin, Chien-Wei Lee, Yi-Hsuan Ho, Huan-Hsien Kuo, Alice L. Yu, John Yu

**Affiliations:** 1 Institute of Stem Cell and Translational Cancer Research, Chang Gung Memorial Hospital at Linkou, and Chang Gung University, Taoyuan, 333 Taiwan; 2 Department of Chemistry, National Tsing Hua University, Hsinchu, Taiwan; 3 Department of Pediatrics, University of California in San Diego, San Diego, CA USA; 4 Institute of Cellular and Organismic Biology, Academia Sinica, Taipei, Taiwan

**Keywords:** Globo H ceramide, TRAX, Phospholipase Cβ1, Angiogenesis, Glycosphingolipids, Extracellular vesicles

## Abstract

**Background:**

In endothelial cells, phospholipase C (PLC) β1-activated Ca^2+^ is a crucial second messenger for the signaling pathways governing angiogenesis. PLCβ1 is inactivated by complexing with an intracellular protein called translin-associated factor X (TRAX). This study demonstrates specific interactions between Globo H ceramide (GHCer) and TRAX, which highlight a new angiogenic control through PLCβ1 activation.

**Methods:**

Globo-series glycosphingolipids (GSLs), including GHCer and stage-specific embryonic antigen-3 ceramide (SSEA3Cer), were analyzed using enzyme-linked immunosorbent assay (ELISA) and Biacore for their binding with TRAX. Angiogenic activities of GSLs in human umbilical vein endothelial cells (HUVECs) were evaluated. Molecular dynamics (MD) simulation was used to study conformations of GSLs and their molecular interactions with TRAX. Fluorescence resonance energy transfer (FRET) analysis of HUVECs by confocal microscopy was used to validate the release of PLCβ1 from TRAX. Furthermore, the in vivo angiogenic activity of extracellular vesicles (EVs) containing GHCer was confirmed using subcutaneous Matrigel plug assay in mice.

**Results:**

The results of ELISA and Biacore analysis showed a stable complex between recombinant TRAX and synthetic GHCer with Kd of 40.9 nM. In contrast, SSEA3Cer lacking a fucose residue of GHCer at the terminal showed ~ 1000-fold decrease in the binding affinity. These results were consistent with their angiogenic activities in HUVECs. The MD simulation indicated that TRAX interacted with the glycan moiety of GHCer at amino acid Q223, Q219, L142, S141, and E216. At equilibrium the stable complex maintained 4.6 ± 1.3 H-bonds. TRAX containing double mutations with Q223A and Q219A lost its ability to interact with GHCer in both MD simulation and Biacore assays. Removal of the terminal fucose from GHCer to become SSEA3Cer resulted in decreased H-bonding to 1.2 ± 1.0 by the MD simulation. Such specific H-bonding was due to the conformational alteration in the whole glycan which was affected by the presence or absence of the fucose moiety. In addition, ELISA, Biacore, and in-cell FRET assays confirmed the competition between GHCer and PLCβ1 for binding to TRAX. Furthermore, the Matrigel plug assay showed robust vessel formation in the plug containing tumor-secreted EVs or synthetic GHCer, but not in the plug with SSEA3Cer. The FRET analysis also indicated the disruption of colocalization of TRAX and PLCβ1 in cells by GHCer derived from EVs.

**Conclusions:**

Overall, the fucose residue in GHCer dictated the glycan conformation for its complexing with TRAX to release TRAX-sequestered PLCβ1, leading to Ca^2+^ mobilization in endothelial cells and enhancing angiogenesis in tumor microenvironments.

**Supplementary Information:**

The online version contains supplementary material available at 10.1186/s12929-022-00889-w.

## Background

Angiogenesis begins with the increase of the intracellular Ca^2+^ level in endothelial cells, which drives their proliferation, differentiation, and tube formation. The most common signaling pathway that increases the cytoplasmic Ca^2+^ concentration is the phospholipase C (PLC) pathway [1]. PLCs hydrolyze membrane phosphatidylinositol-4,5-biphosphate (PIP_2_) into diacylglycerol and inositol-1,4,5-triphosphate (IP_3_), which serve as second messengers. While diacylglycerol remains in the membrane, IP_3_ is released as a soluble factor which diffuses through cytosol to bind the IP_3_ receptor on endoplasmic reticulum (ER), triggering a rapid increase of Ca^2+^ concentration and a cascade of intracellular activities [2–5]. Among various isotypes, PLCγ has been well characterized by its interactions with kinase insert domain receptor (KDR) and fibroblast growth factor (FGF) receptor 1 in endothelial cells, thus participating in the enhancement of angiogenesis [6]. In addition to PLCγ, PLCβ is activated by Gαq protein on the inner surface of plasma membrane, where it catalyzes the PIP_2_ hydrolysis. While PLCβ1 is mainly localized on the cytosolic side of plasma membrane, a substantial fraction is also found in cytosol and binds to a cytosolic translin-associated factor X (TRAX) with high affinity [7]. Thus, TRAX competes with plasma-membrane bound Gαq for PLCβ1 binding, stabilizing PLCβ1 in cytosolic compartment and reducing its activation by Gαq for angiogenesis. Thus, high level of TRAX will eliminate PLCβ activation and quench Gαq-mediated calcium signals in cells [7]. TRAX was known to interact with the C-terminal region of PLCβ1, which is specific to the PLCβ family since this region is absent from any other PLC family members (i.e., γ, δ, ε, ζ, and η). As a multifunctional cytosolic protein, TRAX interacts with binding partners but the details of regulatory controls for PLCβ1 by other TRAX binding partners are unknown.

Glycosphingolipids (GSLs) are a heterogeneous class of membrane glycolipids consisting of a ceramide backbone covalently linked to a glycan moiety and are involved in many cellular processes [8–10]. Many reports suggest that GSLs display diverse functions during embryogenesis and differentiation [8, 9]. Previously, we showed that the expression of globo-series GSLs such as Globo H ceramide (GHCer) (Fucα1 → 2Galβ1 → 3GalNAcβ1 → 3Galα1 → 4Galβ1 → 4Glcβ1-ceramide) [11] and stage-specific embryonic antigen-3 ceramide (SSEA3Cer) (Galβ1 → 3GalNAcβ1 → 3Galα1 → 4Galβ1 → 4Glcβ1-ceramide) was restricted to a brief period of time during early embryonic development [12–14]. These complex GSLs were generally not found in the mature cells of normal tissues, but expressed in cancer cells [12]. In particular, GHCer was reported to be overexpressed in many epithelial cell tumors, such as breast, colon, and lung cancers [15, 16], and is currently used as a target for novel anticancer therapy [17, 18].

However, the biological roles of these tumor associated GSLs in tumor microenvironment have remained elusive. Previous studies indicated that GHCer was not only involved in the suppression of T and B cell activation [8, 9, 18–20] but also induced the activation of human umbilical vein endothelial cells (HUVECs) in vitro and in vivo [17, 21]. In addition, GHCer expression in breast cancer specimens and patient-derived xenograft (PDX) animal model correlated with the microvascular density as measured by anti-CD31 [21]. Studies of immunoprecipitation with anti-GHCer monoclonal antibody (mAb), VK9, followed by mass spectrometry and fluorescence resonance energy transfer (FRET) studies [22], indicated the association of GHCer with the intracellular TRAX in HUVECs [21]. Although these cellular studies imply that GHCer and TRAX exist as a complex, it remains unclear whether they interact specifically as a complex at molecular level and how the complex leads to the enhanced angiogenesis.

Both molecular docking and molecular dynamics (MD) simulation had been used to predict the TRAX–GHCer complex model [23]. However, in the model predicted by the molecular docking, the fatty acid chain of GHCer was found not to have access to the hydrophobic region, which was not consistent with the finding based on the MD simulation [23]. When compared with the molecular docking, the MD simulation is a better method which can generate a stable model with more H-bonds between GHCer and TRAX [23]. Therefore, to investigate the GHCer binding site on TRAX in details, we applied MD method to produce more conformations of GHCer in new molecular docking reported in this study. By comparing with SSEA3Cer, we carried out studies to dissect the contribution of the fucose residue of GHCer in its interaction with TRAX by Biacore and MD simulation. We further validated the molecular interaction by demonstrating the impacts of mutations at key amino acid residues in TRAX. Finally, both synthetic GHCer and the GHCer from tumor-secreted extracellular vesicles (EVs) were validated to compete with PLCβ1 for complexing with TRAX and induce angiogenesis in vitro and in vivo.

## Materials and methods

### Protein expression and purification

Total mRNA was isolated from HUVECs with TRI Reagent (Thermo Fisher Scientific, Waltham, MI, USA); and cDNA was generated from the mRNA with Hight-Capacity cDNA Reverse Transcription Kit (Thermo Fisher Scientific) according to the manufacturer’s protocol. Human full-length TRAX [24] and the C-terminal domain of PLCβ1 (PLCβ1-C) [25] were expressed with N-terminal modifications. The cDNA fragments encoding the modified TRAX and PLCβ1 were prepared from HUVECs and produced by PCR using the primers (TRAX F′-primer: AAGAATTCGAATGAGCAACAAAGAAGGATCAGG; R′-primer: TTAAGCTTCTAGTGGTGGTGGTGGTGGTGGTGGTGGTGAG AAATGCCCTC TTCTTGATC; PLCβ1C F′-primer: AAGGATCCGA GCACCTGCCAAAACAGAAG; R′-primer: TTAAGCTTCTAGTGGTGGTG GTGGTGGTGGTGGTGGTGGATCTTTCCTTTCATGGCTTC) and inserted into the EcoRI/HindIII and BamHI/HindIII-digested pET-21d (Addgene, Watertown, MA, USA) expression vectors, respectively. The F′-primers were designed to add nine histidine residues at the N-terminus to facilitate purification. The expression plasmids were introduced into *Escherichia coli* strain BL21 (DE3) pLys (NEB, Ipswich, MA, USA).

To produce the plasmid pPET-21d-TRAX^Q219A, Q223A^ for mutation experiment, site-directed mutagenesis was carried out using standard full plasmid amplification by DpnI-based PCR strategy with KAPA HiFi HotStart DNA polymerase (Roche, Basel, Switzerland) using pET-21d-TRAX as template. In all cases, fidelity of the amplified DNA was verified by DNA sequencing.

The human recombinant wild-type TRAX, the TRAX^Q219A, Q223A^ protein, and the PLCβ1-C were expressed and extracted from *E. coli*, and purified by ÄKTA pure chromatography system (Cytiva, Marlborough, MA, USA). Extract was centrifuged and the soluble fraction was applied to a HisTrap FF column (5 mL) (Cytiva) equilibrated with buffer A (50 mM potassium phosphate, pH 7.4, 500 mM sodium acetate, 20% glycerol, 0.1 mM EDTA, 0.1 mM dithiothreitol, 1% sodium cholate, 1% Tween 20 and 0.1 mM phenylmethanesulfonyl fluoride); and then the column was washed with 75 mL of buffer A containing 40 mM imidazole and with 20 mL of buffer B (50 mM potassium phosphate, pH 7.4, 20% glycerol, 0.1 mM EDTA, 0.1 mM dithiothreitol, 40 mM imidazole, 1% sodium cholate, 1% Tween 20, 0.1 mM ATP, and 0.1 mM phenylmethanesulfonyl fluoride). Proteins were eluted with buffer C (200 mM imidazole acetate, pH 7.4, 20% glycerol, 0.1 mM EDTA, 0.1 mM dithiothreitol, 1% sodium cholate, and 1% Tween 20). The eluted fractions were combined and diluted with five volumes of buffer D (20 mM potassium phosphate, pH 7.4, 20% glycerol, 0.1 mM EDTA, 0.1 mM dithiothreitol, and 1% sodium cholate) and applied to a DEAE-Sepharose column (30 × 50 mm, Cytiva) equilibrated with buffer E (20 mM potassium phosphate, pH 7.4, 20% glycerol, 0.1 mM EDTA, 0.1 mM dithiothreitol, 10 mM imidazole, 1% sodium cholate, and 0.1% Tween 20). Finally, the column was washed with 40 mL of buffer E. Pass-through fractions were then applied to an SP-Sepharose Fast Flow column (30 × 40 mm) (Cytiva) equilibrated with buffer F (20 mM potassium phosphate, pH 7.4, 20% glycerol, 0.1 mM EDTA, 0.1 mM dithiothreitol, 10 mM imidazole, and 1% sodium cholate). The column was washed with 20 mL of buffer F and eluted with a 0–125 mM NaCl gradient in buffer G (40 mM potassium phosphate, pH 7.4, 20% glycerol, 0.1 mM EDTA, 0.1 mM dithiothreitol, 10 mM imidazole, and 1% sodium cholate). The major eluted fractions were combined, repeatedly concentrated, and diluted using a centrifugal tube (Vivaspin 20) (Cytiva) to replace the buffer with buffer H (50 mM potassium phosphate, pH 7.4, 20% glycerol, 0.1 mM EDTA, 0.1 mM dithiothreitol, 1% sodium cholate, and 0.05% Tween 20).

### GSLs and antibody

GHCer was prepared by modification of previously reported methods [26]. In brief, lactosyl sphingosine was assembled with required sugars by enzymatic synthesis to give Globo H-Sph, and SSEA3-Sph which were coupled with fatty acid, respectively, to yield GHCer (molecular weight: 1535.81) and SSEA3Cer (molecular weight: 1389.67).

Galactosylceramide, lactosylceramide, and Gb4-ceramide were purchased from Matreya LLC (State College, PA, USA). All GSLs in experiments were dissolved in PBS and sonicated right before use. The mAb VK9 hybridoma which was kindly provided by Dr. Ragupathi, was used to produce anti-GHCer antibody [22].

### Enzyme-linked immunosorbent assay (ELISA)

The wells of microtiter plate (Nunc Maxisorp™) (Thermo Fisher Scientific) were coated with 50 μL of GHCer (10 μg/mL in ethanol) or TRAX (10 μg/mL in PBS), followed by incubation overnight at 4 °C. The plate was washed three times with PBS and blocked with 200 μL/well of 1% BSA in PBS for 1 h at room temperature. Empty wells were also blocked as controls. The plate was washed again and incubated with 50 μL/well of TRAX (10 μg/mL to 15.6 ng/mL) at GHCer coated-wells or GHCer (2 μg/mL to 15.6 ng/mL) at TRAX coated-wells for 1 h at room temperature. The plate was washed three times with PBS-T (0.05% Tween 20) and incubated with anti-TRAX antibody or anti-PLCβ1 for 1 h at room temperature. The plate was washed three times with PBS-T before addition of alkaline phosphatase (AP)-conjugated goat anti-mouse IgG diluted 1/4000 in PBS-T and incubation for 45 min at room temperature. The plate was washed again with PBS-T and incubated for 20 min at 37 °C with 100 μL/well of substrate pNPP (Sigma-Aldrich, Darmastadt, Germany). The reaction was stopped with 50 μL of 3 N NaOH and the optical density was measured at 420 nm.

### Biacore assays

Biacore assays for protein-glycan and protein–protein interactions were performed using Biacore X platform (Cytiva). Generally, proteins were immobilized using a standard amine-coupling protocol provided by the manufacturer. The carboxylic acid groups on CM5 chips (Cytiva) were first activated using a mixture of EDC and NHS solutions (Cytiva) at 25 °C for 7 min at a flow rate of 10 μL/min. Subsequently, about 5 μg of recombinant TRAX protein dissolved in 100 μL of sodium acetate buffer (10 mM; pH 5.0) was injected, resulting about 2500-RU responses of proteins which were covalently immobilized on the chip. Finally, the chip was deactivated by ethanolamine and applied to the Biacore X system for binding assays. HBS-P buffer (Cytiva) was used as the running buffer; a 1-min pulse of 0.005% (w/v) sodium dodecyl sulfate dissolved in the running buffer was used to regenerate the chip surface. GSLs were dissolved in methanol as stock solutions (1 mM) and were diluted with HBS-P buffer before Biacore assays. Binding affinities determined by Biacore system were calculated using BIAevaluation Software (version 4.1) (Cytiva).

### Cell culture of HUVECs

HUVECs were obtained from Lonza and maintained in EGM-2 medium (Lonza, Basel, Switzerland) supplemented with 5% fetal bovine serum (Gibco, Waltham, MI, USA), 3 ng/mL bFGF, 5 U/mL heparin, 100 U/mL penicillin, and 0.1 mg/mL streptomycin (Gibco). All cells were cultured for less than 10 passages after resuscitation for experiments.

### Measurement of intracellular free calcium mobilization

Intracellular free calcium mobilization in HUVECs was determined with the fluorescent calcium indicator fluo-4/AM (Thermo Fisher Scientific) [27]. Briefly, 4 × 10^4^ HUVECs were seeded into 96-well black plates with clear bottom plates (Corning, NY, USA) and loaded with fluo-4/AM (40 μM) (Thermo Fisher Scientific) in serum-free EGM-2 at 37 °C for 30 min. The cells were then washed twice with calcium-free Locke solution (158.4 mM NaCl, 5.6 mM KCl, 1.2 mM MgCl_2,_ 0.2 mM EGTA, 5 mM HEPES, and 10 mM glucose, pH 7.3) to remove extracellular dye. Probenecid (2.5 mM) (Sigma-Aldrich) was added to both the loading medium and the washing solution to prevent dye leakage. Cells were exposed to 60 μM GHCer or SSEA3Cer. Fluorescence intensity was determined with a Victor3 (Perkin Elmer, Waltham, MA, USA) using an alternative wavelength time scanning method.

### Tube formation assay

HUVECs were cultured for 4 h under serum-free conditions. Then, growth factor reduced Matrigel (BD Discovery Labware, Bedford, MA, USA) was placed in 12 well plates (Corning) and allowed to polymerize for 30 min at 37 °C. Next, 6 × 10^4^ cells per well were seeded on Matrigel and stimulated overnight in the presence of GHCer, SSEA3Cer, or PBS. Images were taken under microscope (LECIA DFC310 FX, Wetzlar, Germany) in 5–8 fields per well and analyzed by Image J software (National Institute of Health, Bethesda, MA, USA). By using Angiogenesis Analyzer (Gilles Carpentier), a toolset of Image J software, tube area, total length of tube-like structures, number of branches, and number of meshes were quantified on phase contrast images of HUVECs after 16 h of culture.

### Real-time PCR

Total RNA from the cells was purified with a TRIzol Reagent (Thermo Fisher Scientific) following the manufacturer’s instruction. The purity and quantity of the RNA were measured with a spectrophotometer, Nanodrop (Thermo Fisher Scientific). RNA was subjected to high-capacity cDNA reverse transcription kit (Applied Biosystems, Waltham, MA, USA). The cDNA product was amplified and quantified with 7500 Real-time PCR system (Applied Biosystems) using SYBR^®^ Green PCR Master Mix (Applied Biosystems). The primer sets used for PCR amplification were listed below: Vascular endothelial growth factor (VEGF) A F′-primer: TTGCCTTGCTGCTCTACCTCCA; R′-primer: GATGGCAGTAGCTGCGCTGATA. KDR F′-primer: GGAACCTCACTATCCGCAGAGT; R′-primer: CCAAGTTCGTCTTTTCCTGGGC. FGF13 F′-primer: GGGTCAAACTCTTCGGCTCCAA; R′-primer: GGTGCCATCAATGGTTCCATCC FGF2 F′-primer: AGCGGCTGTACTGCAAAAACGG; R′-primer: CCTTTGATAGACACAACTCCTCTC. The thermal cycling program consisted of 2 min at 50 °C, 10 min at 95 °C, followed by 40 cycles for 15 s at 95 °C, and 1 min at 60 °C. The value was normalized with the ratio of mRNA of the target gene to mRNA of the internal reference gene, GADPH, in each sample. Fold change was calculated as the ratio of the normalized values of the cells as compared to the PBS treatment.

### Molecular docking

To generate TRAX-GSL binding modes, molecular docking of GSLs against TRAX was performed using Dock (version 5.1.1; https://dock.compbio.ucsf.edu) software [28]. Subsequently, HotLig [29] software was used to evaluate TRAX–GSL interactions. To predict the binding pocket for GSLs, the cavities on TRAX were first detected using PscanMS [29] software, then these cavities were subjected to analysis using Dock software for docking calculations. Kollman partial charges were applied to protein models for the force field calculations with Dock. Structures of GSLs were first created using Marvin software (version 5.2.2; https://chemaxon.com/marvin) (ChemAxon, Budapest, Hungary) and the three-dimensional coordinates with energy optimization were built using Balloon software (version 0.6; http://users.abo.fi/mivainio/balloon/index.php) [30]. Additionally, the Gasteiger partial charges on the atoms of GSLs were calculated using OpenBabel (version 2.2.3; http://openbabel.org/wiki/Main_Page) [31]. In addition, we also used MD simulation (see below) to generate multiple conformations of GHCer beforehand for docking with TRAX. The parameters for Dock were set to generate 2000 orientations and 200 conformers per cycle of conformational search in the binding pocket with the parameter of “anchor size” set to 1. The GSL conformers docked to TRAX were then scored and ranked by HotLig [29] to predict molecular interactions between GSLs and TRAX. The figures for molecular modeling and interactions were generated using Chimera (version 1.16; https://www.cgl.ucsf.edu/chimera) [32].

### MD simulations

Studies of MD were performed using GROMACS (version 4.5.7-1; https://www.gromacs.org) [33] run on a CentOS (release 6.5; https://www.centos.org) Linux system. We used CHARMM27 force field for the generation of topologies for protein structures, processing of energy minimization, and MD simulations. Structures of GSLs were built using doGlycans packages (https://bitbucket.org/biophys-uh/doglycans) [34]; the topologies with CHARMM force fields were generated using SwissParam (https://www.swissparam.ch) [35]. Subsequently, the GSLs or the protein-GSL complexes were solvated with water using the TIP3P model defined in GROMACS, forming an in-solution system for simulation. Sodium ions were then added to neutralize the system according to the charges of the complexes.

First, the molecular structures in the system were refined by an energy minimization process until the maximum force lower than 100 kJ/mol/nm. Position restrained MD were performed for 20 ps to equilibrate the distribution of the water molecules. Subsequently, MD for the whole system were simulated at a temperature of 300 K, with 500 steps per ps for simulation of atomic motion. The coordinates of molecules were written to trajectory files every 2 ps for analysis of conformational changes and molecular interactions. VMD software (http://www.ks.uiuc.edu/Research/vmd/) was used for making movies of molecular trajectories.

### FRET assay

We examined FRET analysis by confocal microscope (Leica) to study the interaction of PLCβ1 and TRAX [36]. Briefly, HUVECs were incubated with or without 20 μM GHCer or SSEA3Cer for 3 h, 100 μg EVs, EVs + mAb VK9 (5 μg), or EVs + isotype antibody for 16 h, at 37 °C then washed twice in cold PBS. After fixation and permeabilization, cells were stained for PLCβ1 with unlabeled mouse antibody (Santa Curz, Dallas, TX, USA) and a saturating amount of donor Alexa488 labeled anti-mouse IgG (Biolegend, San Diego, CA, USA). TRAX was detected by rabbit anti-TRAX antibodies (Abcam, Cambridge, UK), followed by saturating concentrations of acceptor Alexa555-tagged anti-rabbit IgG (Biolegend). After staining, the cells were washed and analyzed under a confocal microscope (Leica) with 555 laser power-off.

### Immunohistochemistry

Human clinical breast cancer specimens were obtained from patients at the time of initial surgery and were fully encoded to protect patient confidentiality. Clinical specimens were utilized under a protocol approved by the Institutional Review Board of the Human Subjects Research Ethics Committee of Chang Gung Memorial Hospital. For GHCer staining, tissue sections were deparaffinized followed by antigen retrieval by autoclaving at 121 °C for 5 min in AR-10 solution (Biogenex, Fremont, CA, USA). Slides were incubated with mAb VK9 (1:100 antibody dilution buffer) (Ventana Medical Systems, Inc., Oro Valley, AZ, USA) overnight at 4 °C, followed by antibody detection using a polymer-HRP IHC detection system (Biogenex). The slides were counter stained with hematoxylin and mounted. Digital images were captured using an Aperio ScanScope XT Slide Scanner (Aperio Technologies, Vista, CA, USA) under 20× magnification. The expression of Globo H and the morphology of tumor blood vessels were confirmed by pathologists.

### EV isolation, quantitation, and characterization

MCF-7 (Bioresource Collection and Research Center, Hsinchu, Taiwan) cells were cultured and grown in 15 cm dishes (Corning). When cells reached approximately 80% confluence, cells were washed with phosphate buffered saline (PBS) (Gibco) and transferred to fresh media containing 1% EV-free serum (Gibco) for 24 h. Media was then collected and subjected to centrifugation at 500×*g* for 5 min to pellet cells, then 2000×*g* for 15 min to remove cellular debris (Eppendorf, Hamburg, Germany). The supernatant was then transferred to ultracentrifuge tubes (Beckman Coulter, Pasadena, CA, USA) and ultracentrifuged (Beckman Coulter) at 10,000×*g* for 30 min twice to pellet and remove large vesicles. The supernatant was then transferred to new ultracentrifuge tubes and ultracentrifuged twice at 100,000×*g* for 60 min to pellet EVs. EVs were resuspended in PBS and then stored at 4 °C. Protein content of EVs was determined by BCA (Thermo Fisher Scientific) and treatment dosing was determined by EV protein concentration. Particle size was measured using qNano Gold (Izon, Science Ltd., Burnside, New Zealand) by Tunable Resistive Pulse Sensing. Data was recorded and analyzed using Izon Control Suite Software (version 2.2.2.111). Particle size reported are representative of three separate measurements of EVs.

### Transmission electron microscopy and immunogold labelling

EV samples were fixed by adding an equal volume of 4% paraformaldehyde (Merck KGaA, Darmstadt, Germany) to the EV suspension and incubated at room temperature for 10 min. Then, 5 µL of EV suspension were added to carbon-coated 300-mesh copper grids (EMS, Hatfield, PA, USA) for 20 min. Grids were then floated on 100 µL drops of PBS on parafilm for 2 min. Grids were then transferred to 50 µL drops of 1% glutaraldehyde (Merck KGaA) for 5 min. Next, grids were washed on 100 µL drops of PBS for a total of 8 washes and 2 min per wash. Grids were then transferred to 50 µL drops of uranyl oxalate (Sigma-Aldrich), pH = 7.4, for 5 min, and finally to 50 µL drops of 2% methylcellulose (Sigma-Aldrich) and 4% uranyl acetate (EMS) for 10 min on ice. Excess fluid was then blotted off on filter paper; and grids were dried at room temperature for 20 min prior to imaging or storage. Immunolabelling was performed by mounting the concentrated samples on carbon-coated, glow discharged 300 mesh Ni grids (EMS) for 30 s and washed 3 times with PBS. Grids were blocked with 0.5% bovine serum albumin (BSA) (Sigma-Aldrich) in PBS and incubated with primary mAb VK9, anti-GHCer antibody: anti-SSEA3Cer antibody 1:50 in 0.5% BSA in PBS for overnight at 4 °C. After incubation, grids were washed 3 times with PBS and incubated with secondary antibody goat anti-mouse conjugated with 4 nm colloidal gold (Jackson ImmunoResearch, West Grove, PA, USA) 1:20 in 0.5% BSA in PBS. Samples and secondary antibody were incubated for 1 h at room temperature. The grids were then washed with 3 drops of PBS. The grids were finally washed with 3 drops of PBS before staining with 2 drops of methyl cellulose/uranyl acetate and blotted dry. Images were obtained with a transmission electron microscope (HITACHI HT-7800, Krefeld, Germany).

### EV fluorescent labeling

EVs were labeled with 0.05 µL anti-CD9-FITC, anti-CD63-PE, or anti-CD81-APC (Thermo Fisher Scientific) in 100 µL PBS for 30 min on ice in the dark. To avoid false positive events, all antibodies used were run in PBS alone to ensure antibody clumps were not present. To avoid carry‐over effects between each sample measurement we performed a washing step with filtered double distilled water for 1 min at an increased flow rate of 60 µL/min. Flow cytometry analysis was performed on CytoFLEX LX Flow Cytometer (Beckman Coulter). Data is analyzed by FlowJo™ V10 software (BD, Ashland, OR, USA).

### Matrigel plug angiogenesis in vivo assay

Mice were maintained at the animal facility of Chang Gung University (IACUC number: CGU105-027). Animal studies were conducted by the guidelines for the Care and Use of Laboratory Animals and were approved by the Institutional Animal Care and Use Committee. Balb/c mice (National Laboratory Animal Center, Taipei, Taiwan), weighing 20 ± 2 g at the beginning of the experiment, were housed in a room maintained at 23 °C with a 12-h light–dark cycle. To determine the sample size of animal experiments, we used power analysis assuming (difference in means)/(standard deviation) is > 2.5. A total of 30 male mice were anesthetized by isoflurane (USP TERRELL, Lexington, KY, USA) inhalation and randomized into different groups, and given a subcutaneous injection of Matrigel (500 μL/injection) (BD) with a 27-gauge needle (BD). Matrigel plus PBS served as a negative control. Matrigel containing GHCer, SSEA3Cer, GHCer + mAb VK9, EVs, or EVs + mAb VK9 was the test substance. Five mice were used for each group. After 14 days, all mice were sacrificed; the Matrigel plugs were carefully dissected out and analyzed for hemoglobin content. The Matrigel plugs were weighed and homogenized for 5 to 10 min on ice. Supernatants (50 μL) were mixed with 950 μL Drabkin reagent (Sigma-Aldrich) and incubated at room temperature for 30 min, and the absorbance was read with an ELISA reader at 540 nm. Matrigel plugs were fixed in formaldehyde and embedded in paraffin. The Matrigel sections were deparaffinized followed by hematoxylin and eosin (H&E) staining and visualized using Aperio ScanScope XT Slide Scanner (Aperio Technologies). The capillary structure was counted. All mice in each group were examined.

### Dot-blot assay for determination of GHCer concentration

Supernatants from confluent monolayer cultures of MCF-7 cells (Bioresource Collection and Research Center, Hsinchu, Taiwan) were collected on day 5, centrifuged at 1200×*g* for 10 min, and passed through 0.22 μm filter. The culture medium was concentrated (Eppendorf) before loading into membrane. The PVDF membrane was activated in MeOH and washed in PBS and blocked for 30 min with 3% BSA in PBS. Membrane was incubated with anti-GHCer mAb VK9. After incubation with alkaline phosphatase conjugated anti-mouse IgG, immune-reactive GSL dots were detected by enhanced chemiluminescence reagents (Amersham Pharmacia Biotech, Amersham, UK) and analyzed by Typhoon (Cytiva). The optical density of dots detected by dot-blotting was calculated with ImageQuantTL (Cytiva).

### Statistical analysis

Bioassays were replicated three times. Data analysis for tube formation and expression of mRNA was conducted by one-way ANOVA in GraphPad Prism (version 9) (San Diego, CA, USA).

## Results

### Comparison between GHCer and SSEA3Cer for their ability to bind TRAX and promote angiogenesis

**Fig. 1:**
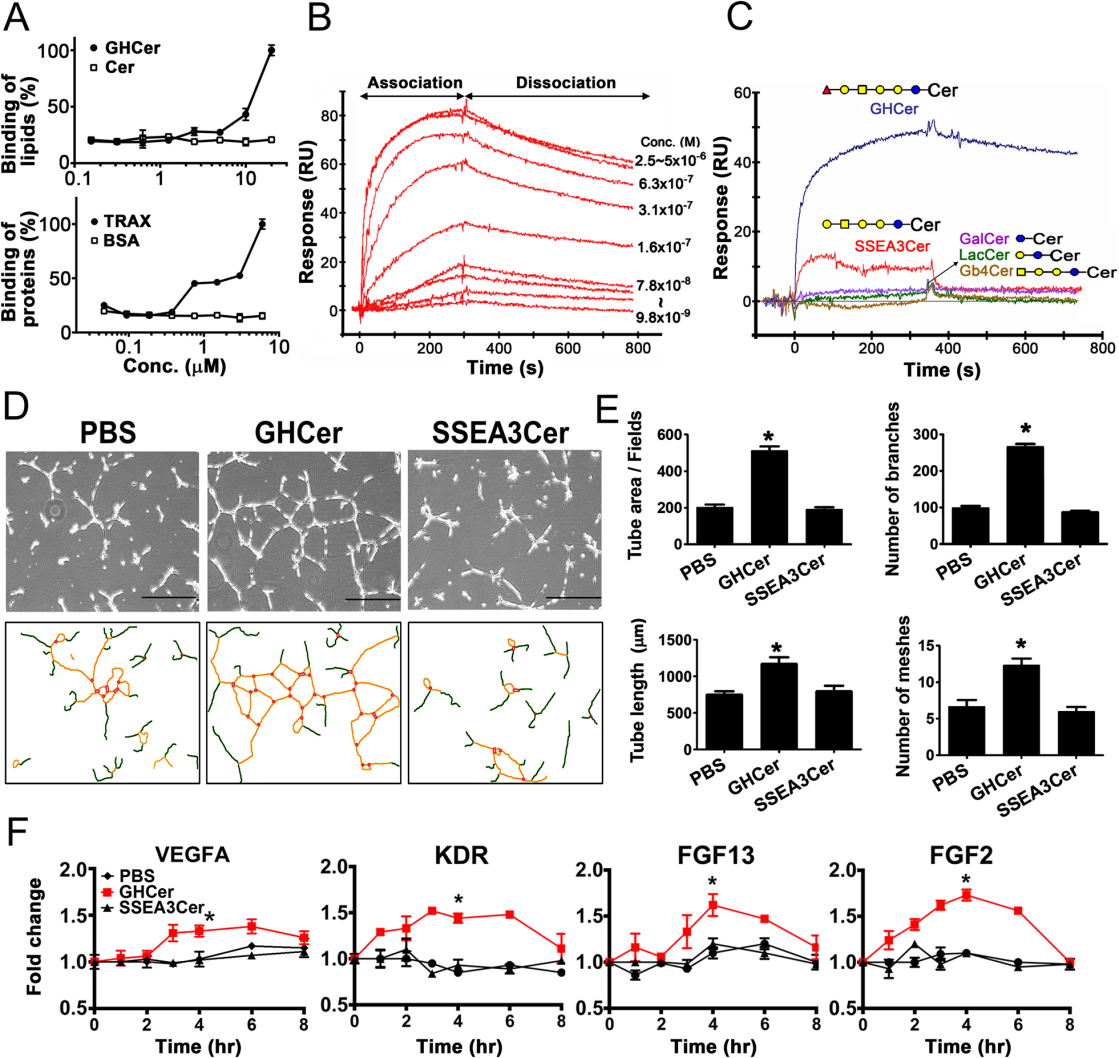
Interaction between GHCer and recombinant TRAX and the increase of angiogenic activities in HUVECs by GHCer. ELISA binding (**A**) and Biacore analysis (**B**) for the binding between GHCer and recombinant TRAX. Recombinant TRAX or synthetic GHCer was immobilized on microtiter plate for ELISA assays and TRAX protein was on CM5 chips for Biacore analysis. **C** Comparison of Biacore assay for the binding between recombinant TRAX and 10 μM of GHCer, or its biosynthetic precursors like SSEA3Cer, Gb4Cer, LacCer, and GalCer. **D** HUVECs were incubated with GHCer, SSEA3Cer or PBS control for overnight and the meshes (orange), branches (green), and junctions (red) of tube formation were observed with phase-contrast microscope (Scale bar = 100 μm). **E** The tube area, total tube length, number of branches, and number of meshes for tube formation were calculated using three random areas of each well. *p < 0.05. **F** HUVECs were incubated with GHCer, SSEA3Cer or PBS for different time and total RNA was extracted for quantitation of *VEGFA, KDR, FGF13 FGF2* by real-time quantitative PCR. The mRNA levels were normalized to *GADPH*. Data were presented as mean ± standard deviation (SD). *p < 0.05

Previously, GHCer was found to be associated with an intracellular protein, TRAX, in HUVECs by immunoprecipitation and FRET studies [21]. But it remains unclear whether they interact directly or indirectly at the molecular level and how their molecular interaction relates to angiogenesis. In this study, we expressed full-length human TRAX in *E. coli* and purified the recombinant TRAX (Additional file 1: Fig. S1). In addition, pure form of GHCer was synthesized by a modified reported method [26]. Figure 1A showed a concentration-dependent binding of the synthetic GHCer to recombinant TRAX immobilized on the ELISA plate, while the binding of the ceramide control lacking the glycan remained low at background level. Similarly, recombinant TRAX also bound to GHCer-coated plate in a dose-dependent manner (Fig. 1A).

The interaction of GHCer with recombinant TRAX immobilized to CM5 chips was also assessed by surface-plasmon resonance-based Biacore assay [23]. This interaction displayed a fast association at 2.5–5 μM in the first 300 s, followed by slow dissociation of GHCer from TRAX, giving rise to the Kd of 4.09 × 10^–8^ M (*p*Kd = 7.39) for the GHCer/TRAX complex (Fig. 1B). To examine the binding specificity, GHCer and its biosynthetic precursors [SSEA3Cer, Gb4-ceramide (Gb4Cer), lactosyl-ceramide (LacCer), and galactosyl-ceramide (GalCer)] were similarly evaluated for TRAX binding by Biacore (Fig. 1C). At 10 μM, only GHCer displayed substantial binding with TRAX while SSEA3Cer showed weak binding (Kd > 1 × 10^–5^ M). In contrast, other biosynthetic precursors such as Gb4Cer, LacCer, or GalCer, did not show any binding. It is noted that the only difference between the structures of GHCer and SSEA3Cer, is the extra α1, 2-fucose residue at the terminus of glycan for GHCer, suggesting an important contribution of fucose to the interaction between GHCer and TRAX.

Next, we compared the angiogenic activity of GHCer and SSEA3Cer in HUVECs. As shown in Fig. 1D, incubation of HUVECs with 20 μM GHCer, but not with SSEA3Cer, increased the tube-formation of the cells. Quantitation with Angiogenesis Analyzer showed that GHCer considerably increased the tube formation in HUVECs as evaluated by tube area, tube-length, branch number, and number of meshes; in contrast, SSEA3Cer did not have significant effects on the tube formation when compared with PBS control (Fig. 1D, E). Furthermore, the addition of GHCer led to a rapid increase of mRNA expression of angiogenesis-related genes in HUVECs including *VEGFA*, *KDR*, *FGF13*, and *FGF2,* which peaked at approximately 4 h of incubation with GHCer (Fig. 1F). But the addition of SSEA3Cer did not affect mRNA expression of these genes.

### In silico analysis of the TRAX–GHCer and TRAX–SSEA3Cer complex

**Fig. 2:**
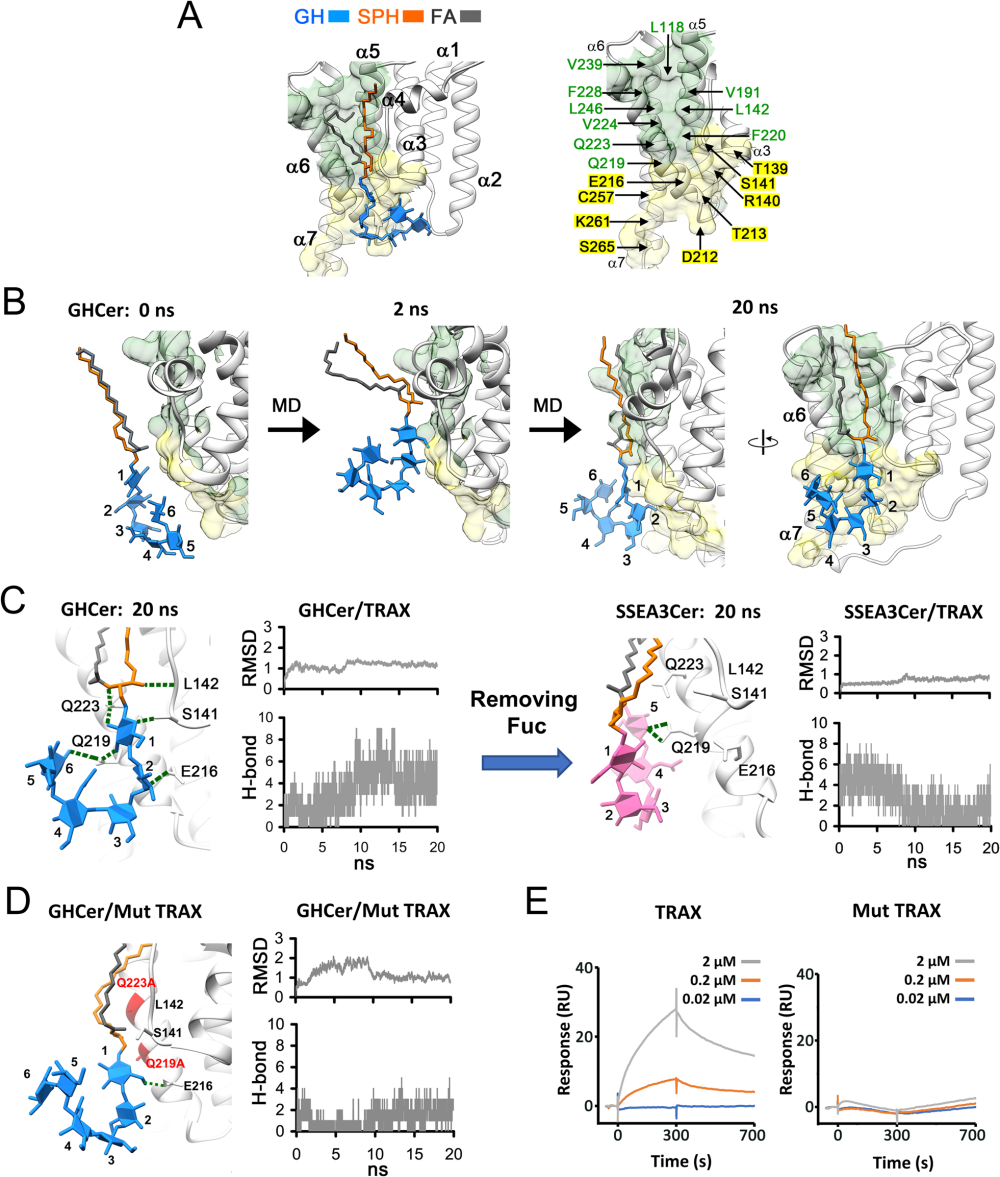
The in silico and Biacore analysis of the molecular interactions within the GHCer/TRAX complex. **A** The TRAX protein had seven alpha helices (α1–α7). The carbon chains of sphingosine (SPH; orange) and fatty acid (FA; black) of GHCer bound to a hydrophobic groove in TRAX (green surface). On the other hand, the glycan of GHCer interacted mainly with a hydrophilic region in TRAX (yellow surface). The amino acid residues in these amphipathic binding sites were labeled. **B** MD simulation was performed for assembling the GHCer/TRAX complex. Various GHCer conformers associated with the binding sites on TRAX by changing its conformation during MD simulation and finally formed a stable complex at equilibrium. The right panel shows the model of stable complex with 90° rotation. **C** The model of GHCer/TRAX complex at equilibrium (20 ns) showed that potentially as many as seven H-bonds could take place between GHCer and TRAX as predicted by MD simulation. When the conformers finally reached equilibrium, the molecular trajectories of MD simulation indicated that the number of H-bonds fluctuated and the average number of H-bonds between GHCer and TRAX was 4.6 ± 1.3. Furthermore, the SSEA3Cer/TRAX complex was generated from GHCer/TRAX model by replacing the Fuc molecule with a hydrogen atom. During MD simulation, the number of H-bonds between SSEA3Cer and TRAX decreased with time due to the conformational changes of SSEA3Cer. **D** MD simulation displayed poor interaction between GHCer and mutant form of TRAX (Mut TRAX with Q223A and Q219A). Only sugar #1 of GHCer formed H-bond with E216 of TRAX. **E** The CM5 chips were coated with mAb VK9 for Biacore assays. GHCer was then captured by the chip, followed by analyzing binding responses of various concentrations of wild-type TRAX or Mut TRAX

The findings that GHCer*,* but not SSEA3Cer, could bind recombinant TRAX implies that such interactions might be specific and fucose-dependent. We performed in silico analysis of binding sites in TRAX for GHCer, based on the X-ray crystallography data showing that TRAX protein (Protein Data Bank entry: 3PJA) contains seven α helices (α1–α7) in the 32–274 amino acid portion of TRAX [24]. As shown in Fig. 2A, molecular docking analysis revealed a hydrophobic groove (green) and a hydrophilic area (yellow) in TRAX as the binding sites for GHCer. The sphingosine (orange) and the fatty acid (black) of GHCer had access to the hydrophobic groove, which comprised of the upper parts of α5 and α6 helices of TRAX containing leucine, valine, phenylalanine (green). On the other hand, the glycan moiety (blue) of GHCer interacted with the hydrophilic surface of TRAX which constituted the lower parts of α3, α5, α6, and α7 helices with polar amino acids, such as glutamic acid, cysteine, lysine, serine, aspartic acid, threonine, and arginine (yellow). It is noted that in this model, glycan of GHCer (blue) pointed outward from the hydrophilic area of TRAX toward the lower part of α3 helix (Fig. 2A).

To investigate the complex assembly between GHCer and TRAX at molecular level, two MD simulations were conducted: the first was used to optimize the conformation of GHCer in-solution at low energy state (see Additional file 2 for the MD movie), and the second simulation assembled the GHCer/TRAX complex to reach equilibrium (Additional file 3). At the beginning of the second MD simulation (0 ns), the optimized in-solution conformer of GHCer was placed in the vicinity of the putative binding site of TRAX initially (Fig. 2B). With time (e.g., 2 ns), GHCer began to associate with TRAX by changing its conformations and finally formed a stable TRAX/GHCer complex at equilibrium after 20 ns (Fig. 2B) (Additional file 3). Similar to the molecular docking analysis (Fig. 2A), MD simulation revealed that sphingosine (orange) and fatty acids (black) of GHCer underwent conformational changes and finally bound to the hydrophobic groove (green, Fig. 2B) of TRAX protein to reach equilibrium. When complexing with TRAX at equilibrium, the MD simulation showed that the glycan (sugars 1 to 6) of GHCer spread beyond the hydrophilic area (yellow). But, unlike the molecular docking complex, the sugars of GHCer extended further outward to the lower parts of α6 and α7 helices after 90° rotation as shown in Fig. 2B (see comparison with Fig. 2A). The model of GHCer/TRAX complex at the 20 ns equilibrium by MD simulation was refined by energy minimization to find potential H-bonds (Fig. 2C). As the atomic distance was less than 4 Å, the H-bond donor/acceptor pairs could generate H-bonding [23]. Thus, GHCer was found to interact with the Q223, Q219, L142, S141, and E216 of TRAX, generating as many as seven H-bonds in the refined complex (green dashed lines in Fig. 2C). Such MD simulation for GHCer/TRAX complex portrayed the root-mean-square deviation (RMSD) curve which is a measure of the conformational changes of GHCer for the complex during the process of reaching a dynamic equilibrium state (Fig. 2C). The number of H-bonds for the assembly of GHCer/TRAX complex fluctuated with time; but finally maintained at 4.6 ± 1.3, when the conformers within the period of 10–20 ns reached stable equilibrium state (Fig. 2C).

To assess the role of fucose residue in GHCer/TRAX complex, MD simulation using SSEA3Cer (without the terminal fucose in the glycan) was performed for comparison. The sugar 6, Fuc, of GHCer was removed (Fig. 2C) to become the SSEA3Cer and then formed complex assembly with TRAX. In 20 ns simulation, the SSEA3Cer/TRAX complex reached equilibrium (see Additional file 4 for the MD movie). While sphingosine and fatty acid of SSEA3Cer were retained in the hydrophobic groove, the glycan of SSEA3Cer dissociated from the hydrophilic binding area of TRAX (i.e., Q223, Q219, L142, S141, and E216) after 20 ns simulation. Notably, at this equilibrium state, SSEA3Cer formed only two potential H-bonds between sugar 5 and Q219 of TRAX protein (Fig. 2C) (Additional file 4). As mentioned, the GHCer/TRAX complex at equilibrium state formed a total of 4.6 ± 1.3 H-bindings. As shown in Fig. 2C (right panel), the molecular trajectories of MD simulation indicated that the number of H-bonds fluctuated and decreased to 1.2 ± 1.0 in 10–20 ns. These results agreed with the data from Biacore and ELISA assays, which support the notion that TRAX preferentially binds GHCer rather than SSEA3Cer.

The results also suggested that Q223 and Q219 amino acids of TRAX played key roles for complexing with GHCer. To validate the contribution of these two amino acid residues to their molecular interactions, we performed similar MD simulation with in-silico model using mutant TRAX (Mut TRAX), which had Q223A and Q219A mutations (Fig. 2D). The resulting GHCer/Mut TRAX complex showed that the fucose (sugar 6) of GHCer was dissociated from Mut TRAX in MD simulation (see Additional file 5). In this model, sugar 1 of GHCer formed one H-bond with E216; but none of the other sugars of GHCer interacted with this mutant form of TRAX (Fig. 2D). The molecular trajectories of the GHCer/Mut TRAX complex showed that the number of H-bonds decreased from 4.6 ± 1.3 to 1.6 ± 0.7, when GHCer and Mut TRAX reached dynamic equilibrium (Fig. 2D).

To further confirm these findings, we constructed a plasmid pPET-21d-TRAX^Q219A, Q223A^ by site-directed mutagenesis and generated the recombinant Mut TRAX protein with the Q223A and Q219A double mutations (Additional file 1: Fig. S1). The Biacore sensorgrams showed that Mut TRAX with Q223A and Q219A double mutations exhibited no binding with GHCer; instead, wild type TRAX bound GHCer in a concentration-dependent manner (Fig. 2E). These findings agreed with the molecular interactions of the GHCer/TRAX complex as predicted by MD simulation. The results also confirmed that the amino acid residues, Q223 and Q219, in TRAX protein played important roles in the binding to GHCer by TRAX.

### GHCer competes with PLCβ1 for binding to TRAX

**Fig. 3:**
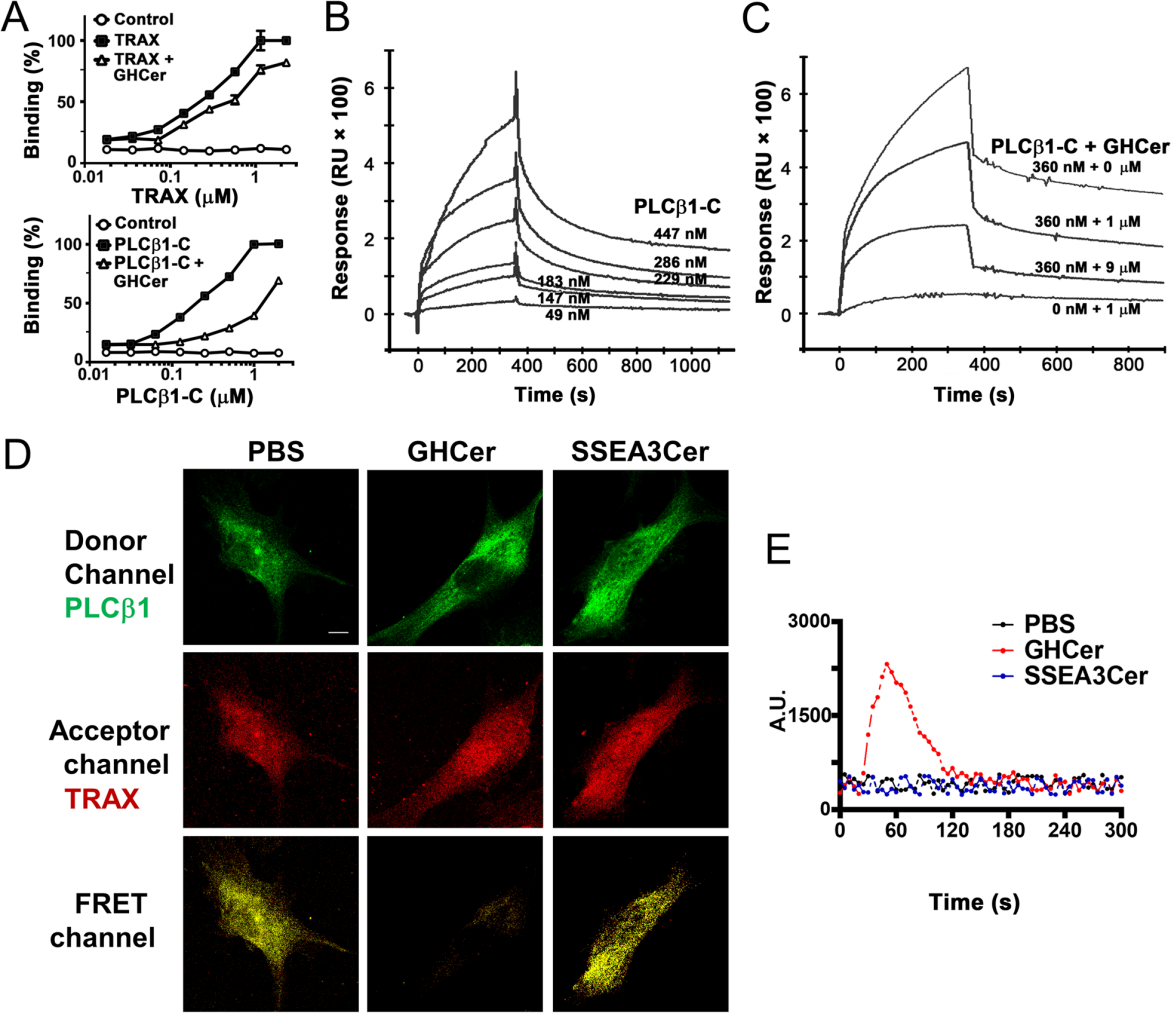
GHCer, but not SSEA3Cer, competed with PLCβ1 for binding to TRAX. **A** Competitive ELISA. Recombinant PLCβ1-C (upper panel) or TRAX (lower panel) protein was immobilized on microtiter plate for ELISA assays. GHCer (20 μM) competed with the binding between TRAX and PLCβ1-C, respectively. The amount of binding protein was determined, respectively, by anti-TRAX and PLCβ1-C antibodies followed by AP-conjugated secondary antibody. BSA protein was used as a negative control. The data were presented with mean ± SD. **B** Biacore analysis of PLCβ1-C binding to TRAX. The TRAX protein was immobilized on CM5 chips. The sensorgram showed that PLCβ1-C bound TRAX in a concentration-dependent manner. The Kd estimated by BIA evaluation was about 7.5 × 10^–6^ M. **C** Biacore analysis for competition between GHCer and PLCβ1-C for binding with TRAX. The CM5 chips coated with TRAX protein were used for binding assay for PLCβ1-C (360 nM), or in combination with 0, 1, and 9 μM of GHCer. **D** FRET assay using confocal microscopy for the colocalization of TRAX and PLCβ1. HUVECs were incubated with 20 μM GHCer or SSEA3Cer for 3 h and treated with mouse anti-PLCβ1 antibody followed by donor Alexa 488-tagged anti-mouse IgG (Green). In addition, the cells were also treated with rabbit anti-TRAX antibody, followed by acceptor (Alexa 555) -tagged anti-rabbit IgG (Red). FRET signal was determined by 555 nm laser power-off. Representative pictures of FRET channel (yellow) from three independent experiments were shown. **E** GHCer-induced calcium influx. After loading with the calcium indicator Fluo-4/AM (40 μM) for 45 min at 37 °C, the fluorescence intensity of intracellular Ca^2+^ in HUVECs was traced for 6 min every 5 s and presented as arbitrary units (a.u.). Experiments were performed upon exposure to PBS, 60 μM of GHCer, or SSEA3Cer. The data are presented as mean ± SD of triplicate determinations

It was reported that binding of TRAX to the C-terminal domain of PLCβ1 prevented PLCβ1 from activation and induction of Ca^2+^ influx to enhance angiogenesis [7]. To examine whether the binding of GHCer to TRAX competes with the interaction of TRAX with the C-terminal domain of PLCβ1, we prepared recombinant C-terminus of PLCβ1 containing amino acid sequences from 890 to 1161 (PLCβ1-C) (Additional file 1: Fig. S1). In ELISA assay, TRAX bound the PLCβ1-C-coated plates in a concentration-depending manner (Fig. 3A). When GHCer was added, the binding of TRAX was greatly reduced. Similarly, the recombinant PLCβ1-C showed a concentration-dependent binding to TRAX-coated plates, which was significantly reduced by the addition of GHCer. These molecular studies indicated that GHCer competed with the PLCβ1-C for interaction with TRAX. These findings were further verified by Biacore assay. The sensorgram showed that the recombinant PLCβ1-C bound to TRAX immobilized on the chip with Kd of ~ 7.5 × 10^–6^ M (Fig. 3B). While the maximum binding of PLCβ1-C at 360 nM for TRAX was 680 response unit (RU), the response was reduced to 470 and 250 RU, with the addition of 1 and 9 μM GHCer, respectively (Fig. 3C). In contrast, SSEA3Cer showed no significant effects on decreasing the binding of PLCβ1-C to TRAX (Additional file 1: Fig. S2). These results confirmed the competition between GHCer and PLCβ1-C for TRAX binding at molecular level.

To demonstrate that the competition between GHCer and PLCβ1 for TRAX binding, which is fucose dependent, occurred at cellular level, we performed FRET analysis using confocal microscopy to study the colocalization of PLCβ1 and TRAX in HUVECs in the presence or absence of GHCer or SSEA3Cer. As shown in Fig. 3D, HUVECs were incubated with PBS, GHCer or SSEA3Cer at 20 μM for 3 h. FRET was then examined using anti-PLCβ1 tagged with Alexa 488 (green) and anti-TRAX with Alexa 555 antibodies (red). As shown Fig. 3D, FRET signal from PLCβ1/TRAX complex was observed in the FRET channel of HUVECs treated with PBS control (yellow color). Meanwhile, TRAX and PLCβ1 expression level remained unchanged during this time period (Additional file 1: Fig. S3) suggesting the colocalization of PLCβ1 and TRAX in cell. However, FRET signal was not detected in HUVECs incubated with GHCer. On the other hand, incubation with SSEA3Cer did not affect the FRET signal for colocalization. These results indicate that at cellular level, GHCer but not SSEA3Cer competed with PLCβ1 for binding to TRAX. We further showed that PLCβ1 displaced by GHCer binding to TRAX could activate calcium influx, the earliest event of signal transduction in angiogenesis [1]. As shown in Fig. 3E, calcium influx in HUVECs rose from 20 to 100 s upon incubation of with GHCer, but not with SSEA3Cer. These findings support the notion that GHCer competes with PLCβ1 for binding to TRAX and enhances angiogenesis, which is fucose dependent.

### GHCer from tumor-secreted EVs induces angiogenesis

**Fig. 4:**
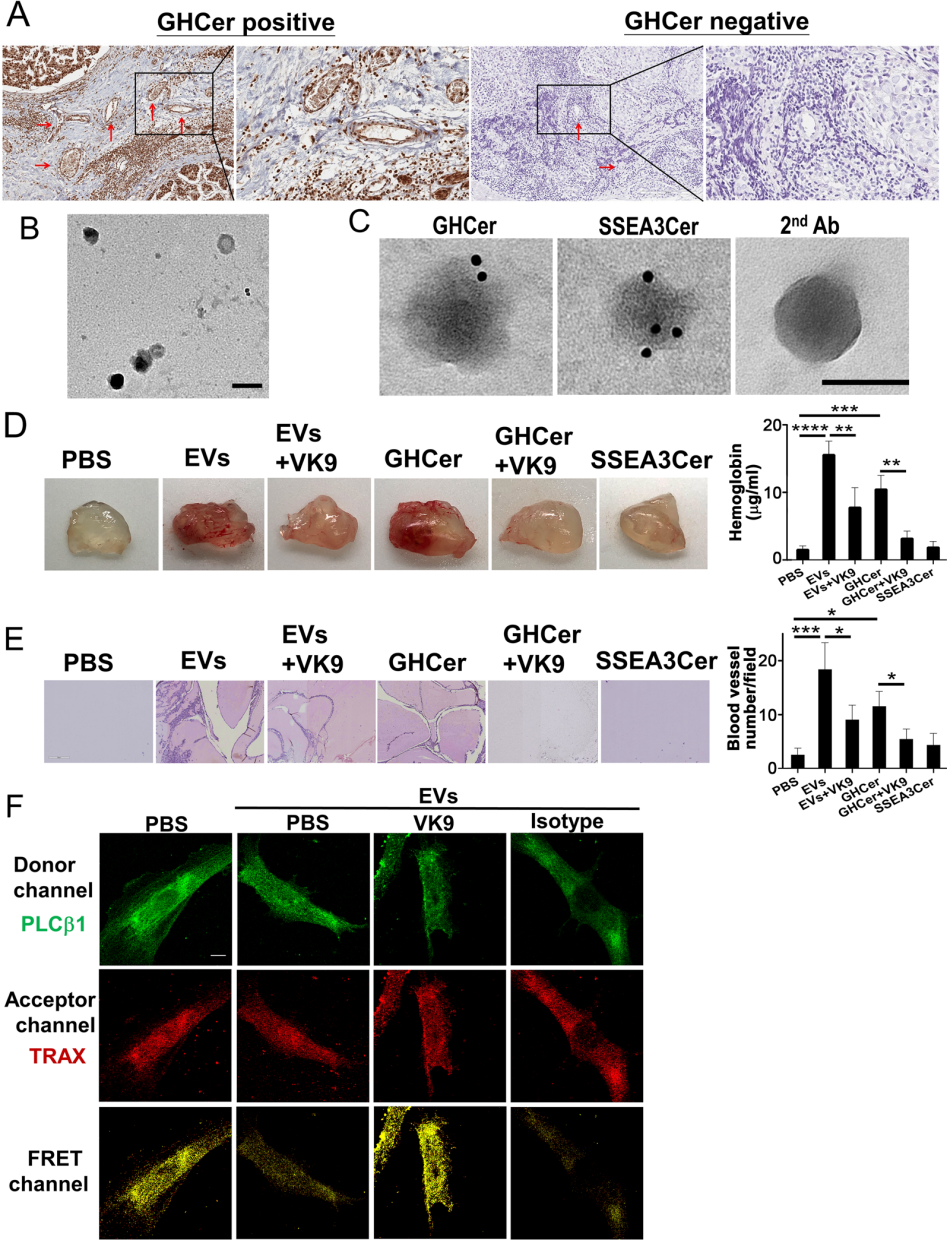
The angiogenesis enhanced by GHCer in EVs competed with PLCβ1 for TRAX binding. **A** Immunohistochemical staining of tissue sections with breast cancer using VK9 mAb. Red arrows indicate vessel formation. **B** Morphology and dimension of the EVs secreted from MCF-7 cells. The EVs were placed on carbon-coated copper grids, stained with uranyl acetate, and examined by transmission electron microscopy. Scale bar: 50 nm. **C** Immunogold staining of the isolated EVs using VK9 or anti-SSEA3Cer antibodies, followed by gold particle-conjugated secondary antibody (2^nd^ Ab). Scale bar: 50 nm. **D** Matrigel (500 μL) mixed with EVs (30 μg), EVs (30 μg) + VK9 (5 μg), GHCer (16.2 μg), GHCer (16.2 μg) + VK9 (5 μg), or SSEA3Cer (14.7 μg) was injected subcutaneously to the flank of mice (n = 5). On day 14, Matrigel plugs were dissected and photographed, and the hemoglobin concentration was determined. **p < 0.01, ***p < 0.001, and ****p < 0.0001. **E** In vivo neovascularization of Matrigel plug induced by GHCer in EVs was examined by H&E staining and quantified for blood vessel density. *p < 0.05 and ***p < 0.001. **F** FRET assay for the colocalization of TRAX and PLCβ1 in HUVECs after incubation with EVs. HUVEC were incubated with PBS, EVs (100 μg), EVs + VK9 (5 μg), or EVs + isotype antibody for 16 h. The result shown here as mean ± SD is one representative from three independent experiments

Previously, we reported that the expression of GHCer in clinical specimens of breast cancer correlated with the increased blood vessel densities [21]. A close examination of the tumor specimens revealed the expression of GHCer not only in the tumor parts per se, but also in the tumor endothelial cells (red arrows in Fig. 4A). Since EVs encapsulating a variety of functional molecules were shown to mediate communications between cells and regulate physiological processes [37–39], it is possible that EVs secreted from cancer cells may mediate the transfer of GHCer to endothelial cells, leading to increased angiogenesis.

We isolated EVs secreted from MCF-7 cells which were characterized to be 70–120 nm in size and expressed CD9, CD63, and CD81 EV markers (Additional file 1: Fig. S4A and B). Under transmission electron microscopy, these EVs displayed a typical round morphology with positive immunogold staining for GHCer and SSEA3Cer (Fig. 4B and C). The in vivo angiogenic activity of these tumor-secreted EVs was assessed by Matrigel plug assay [21]. At 14 days after subcutaneous implantation to mice, formation of new blood vessels was clearly visible in the Matrigel plug containing EVs but not in PBS control (Fig. 4D). The amount of hemoglobin was significantly higher in the plug containing EVs (15.5 ± 1.9 μg/mL, p < 0.0001) than in PBS plug (1.6 ± 0.4 μg/mL). On the other hand, when EVs were pretreated with anti-GHCer antibody (VK9) to neutralize GHCer, the hemoglobin accumulation decreased to 7.8 ± 2.7 μg/mL in the plug (p < 0.01) (Fig. 4D). Since MCF-7 derived EVs contained both GHCer and SSEA3Cer, synthetic GHCer and SSEA3Cer were used in the Matrigel plug assay to determine which GSL species display angiogenic activity. As shown in Fig. 4D, Matrigel plug containing GHCer induced formation of blood vessels and accumulation of hemoglobin (10.4 ± 1.9 μg/mL, p < 0.001); but SSEA3Cer did not (2.0 ± 0.7 μg/mL, p = 0.99) (Fig. 4D). On the other hand, when GHCer was pretreated with VK9, the hemoglobin accumulation decreased to 3.3 ± 1.0 μg/mL in the plug (p < 0.01) (Fig. 4D). In addition, histological examination confirmed the robust vessel formation in Matrigel plug containing tumor-secreted EVs or synthetic GHCer, but not in the plug with PBS control or SSEA3Cer. The quantities of capillary structures were higher in Matrigel plug containing EVs (17.0 ± 4.6, p < 0.001), and GHCer (10.7 ± 2.5, p < 0.05) than those containing PBS (2.3 ± 1.2), but not SSEA3Cer (4.0 ± 2.0, p = 0.94). In contrast, incubation of EVs with VK9 or GHCer with VK9 reduced the blood vessel formation, density was considerably reduced to 8.3 ± 2.5 (p < 0.05), and 5.0 ± 1.7 (p < 0.05), respectively (Fig. 4E). These findings indicate that GHCer in EVs can promote angiogenesis in vivo.

To show that the competition between GHCer and PLCβ1 for TRAX binding was mediated through EVs, we performed another FRET analysis with HUVECs (Fig. 4F). First, HUVECs were incubated for 16 h with PBS or EVs (100 μg) with or without mAb VK9 or isotype control (5 μg). Similar to Fig. 3D, FRET was examined with anti-PLCβ1 tagged with Alexa 488 (green) and anti-TRAX tagged with Alexa 555 (red) (Fig. 4F). While FRET signal from PLCβ1/TRAX complex (yellow) was observed in the FRET channel of PBS control by confocal microscope analysis, the FRET signal was significantly reduced after incubation of HUVECs with EVs. The addition of mAb VK9 to EVs, but not isotype control could restore this FRET signal. (Fig. 4F). These results agreed with the experiments using the synthetic GHCer in Fig. 3D. These experiments using intact cells lend further support that angiogenic activity was mediated through the competition between PLCβ1 and the EV-derived GHCer for TRAX binding [21].

**Fig. 5:**
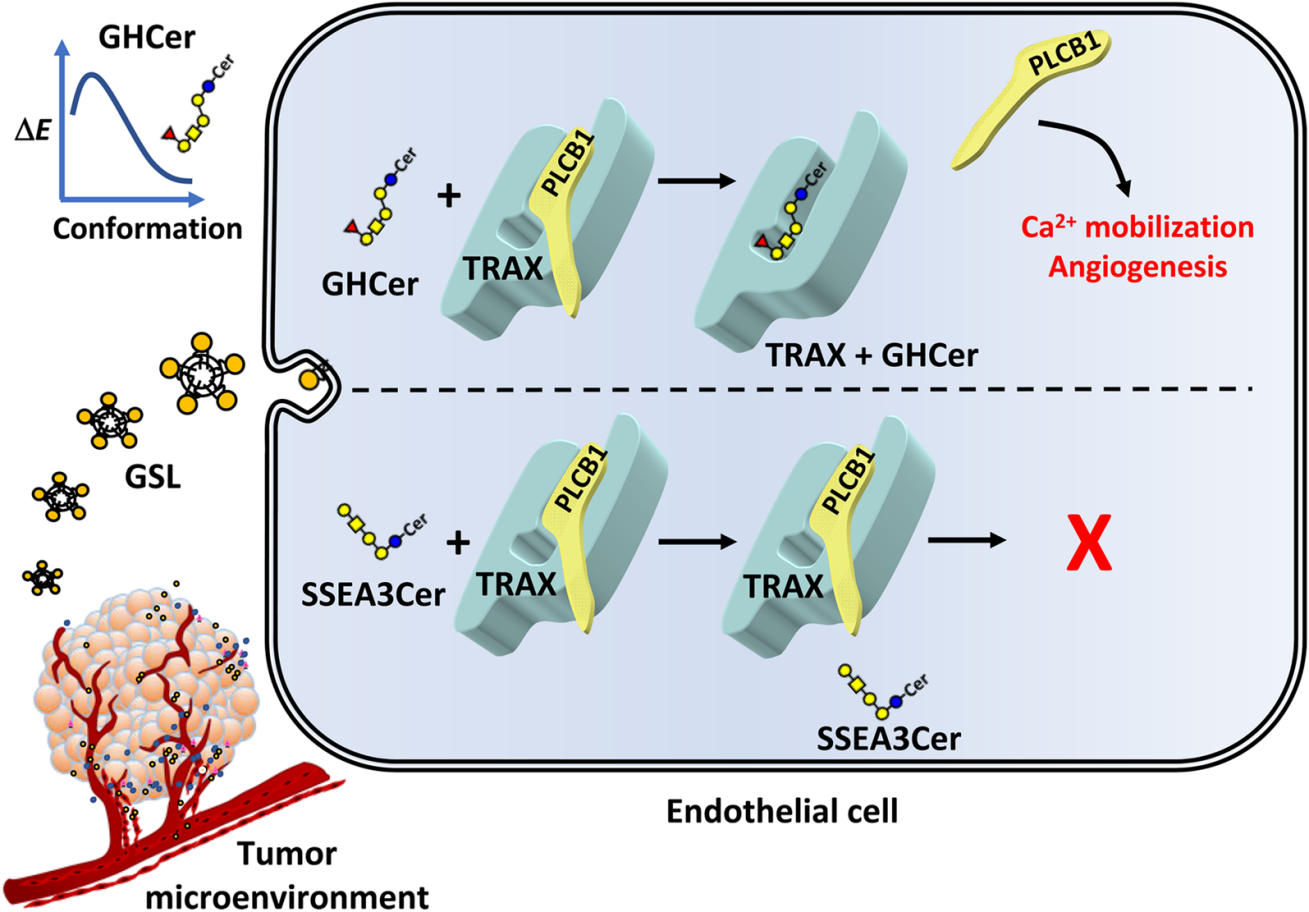
The proposed molecular mechanism for GHCer-induced enhancement of angiogenic activity in tumor microenvironment. The fucose residue of GHCer not only affects its interaction with TRAX at low energy conformation to form stable GHCer/TRAX complex, but also generate several H bondings and hydrophobic interactions with TRAX protein. In addition, GHCer competes with PLCβ1 for binding to TRAX, leading to the dissociation of PLCβ1 to the cytosol and enhancement of angiogenesis in tumor microenvironment

Since the competitiveness between GHCer and PLCβ1 is dose-dependent, it would be of interest to ascertain the concentration of GHCer in the conditioned medium of tumor cells. Using dot-blot analysis, we measured the amount of GHCer in the conditioned medium of MCF-7 cells in confluent culture (Additional file 1: Fig. S4C). By interpolation from the standard curve, the average GHCer concentration in 5-day cultures of MCF-7 cells was 9.1 ± 1.7 μM (Additional file 1: Fig. S4C–F), which far exceeded the K_D_ for GHCer binding to TRAX (40.9 nM). Conceivably, the GHCer concentration in tumor microenvironment could be much higher, as tumor cells were often more densely packed, suggesting that GHCer concentrations in tumor microenvironment might be sufficient to enhance angiogenesis. As illustrated in Fig. 5, a novel molecular mechanism for GHCer-induced enhancement of angiogenesis in tumor microenvironment is proposed: GHCer incorporated into endothelial cells via EVs competes with PLCβ1 for binding to TRAX, leading to the dissociation of PLCβ1 from TRAX to induce Ca^2+^ influx and activation of angiogenesis in tumor microenvironments.

## Discussion

Previously we showed that GHCer co-immunoprecipitated with an intracellular TRAX protein in HUVECs [21]. Here we used Biacore and ELISA assays to demonstrate that synthetic GHCer in pure form, but not its precursor GSLs (including SSEA3Cer), could bind recombinant TRAX with a Kd of 4.09 × 10^–8^ M. In addition, MD simulation revealed stable complex between TRAX and GHCer in which sphingosine and fatty acid chains bound to a hydrophobic groove at α5 to α6 helix of TRAX, and glycan moiety interacted with Q223, Q219, L142, S141, and E216 in hydrophilic area, forming 4.6 ± 1.3 H-bonds at equilibrium state. TRAX containing mutations Q223A and Q219A lost its ability to interact with GHCer. This binding of GHCer–TRAX and the catalytic residues (E126, E129, D193, and E197), which are required for the nuclease activity, are located at the opposite sides of the TRAX molecule [24]; therefore, GHCer may not interfere directly with the nuclease activity of TRAX. In addition, removing the terminal fucose of GHCer yielding SSEA3Cer resulted in decreased H-bonding with TRAX on MD simulation, consistent with Biacore and ELISA findings and failure of SSEA3Cer to promote angiogenesis. Moreover, GHCer competed with PLCβ1 for binding to TRAX in Biacore and FRET assays in HUVECs. These results provided a noble molecular mechanism for GHCer-induced angiogenesis in tumor microenvironments by releasing the TRAX-sequestered PLCβ1 for Ca^2+^ mobilization and enhanced angiogenesis.

Angiogenic cytokines such as VEGF are well-known factors for promoting angiogenesis [40]. Whether biomolecules such as carbohydrates or GSLs could promote angiogenesis in tumor microenvironment has not been thoroughly explored. One such example is the interaction of galectin-1, a secretory galactoside-binding protein induced by hypoxia [41, 42], with the N-glycan part of KDR on cell surface, thereby conferring VEGF-like signaling for tumor-associated angiogenesis [41, 42]. In contrast to such extracellular bindings between galectin-1 and the glycan on receptor, GHCer binds to intracellular cytosolic TRAX, thereby releasing the sequestered PLCβ1, to mobilize Ca^2+^ in endothelial cells and enhance angiogenesis. Subsequently, the GHCer-induced elevation of intracellular Ca^2+^ concentration triggers molecular processes to remove excess cytoplasmic Ca^2+^, so as to maintain Ca^2+^ homeostasis. For instance, the Ca^2+^ release is regulated by phosphorylation of IP_3_ receptor [43]. Calcineurin, an endogenous phosphatase of IP_3_ receptor, which is activated by the increase of the intracellular Ca^2+^ concentration, could then dephosphorylate IP_3_ receptor and terminate the Ca^2+^ release [44]. These molecular processes might denote a new paradigm for promoting angiogenesis in tumor microenvironment.

On the other hand, GSLs are present on the outer surface of plasma membrane. GSLs are conventionally known to interact with membrane components on the surface of the same or the adjacent cells to influence signaling, receptor trafficking, cell–cell contacts and adhesion, and gene expression [8, 19]. For example, the complex assembly between GM3 and epidermal growth factor receptor [45] or insulin receptor [46] at plasma membrane leads to marked changes in their intracellular signaling. Translocation of the membrane-bound GSLs to intracellular site might occur through endocytosis, vesicular trafficking, membrane–membrane contact site formation, or protein binding [23]. The transferred GHCer has been detected in the cytosol of HUVECs in our previous studies, using immunofluorescence confocal microscopy [21]. Upon incubation with HUVECs, GHCer first appeared at the plasma membrane; within minutes, GHCer appeared in the cytoplasm, which increased with time. The transfer of GHCer to cytosol was blocked by an endocytosis inhibitor. Such cytosolic form of GHCer might offer an opportunity for being captured by intracellular protein like TRAX, which possesses amphipathic sites for recognition of GSLs. Similarly, in a few studies, interaction with intracellular components for GSL were reported. For instance, GM1 gangliosides have been found at the inner aspects of nuclear envelope, where they make direct contact with chromatin and influence the activity of promoters for epigenetic activation of neuronal cells [47]. In addition, upon CD95/Fas triggering on cell membrane, the raft-associated GD3 gangliosides become associated with microtubules and localized in mitochondria where they participate in apoptotic stimulation of T cell [48]. Despite the plethora of GSL involvement in many cellular processes on plasma membrane, these recent reports are but a few instances where the mechanisms underlying GSL-induced modulations of cellular functions were reported to occur inside cells.

Both GHCer and SSEA3Cer are tumor-associated GSLs, but they differ in angiogenic activities in that only GHCer but not SSEA3, exhibits proangiogenic activity. Based on the analysis of MD simulations, such distinctive biological activities might be attributed to the unique conformational change in the glycan moiety of GHCer at equilibrium state, which enable the structural recognition of GHCer by TRAX. In addition, the terminal fucose moiety of GHCer seems to play a critical role in generating the glycan conformation suitable for forming H-bonds with TRAX. In comparison, the glycan conformation of SSEA3Cer, which lacks the terminal fucose, was unable to create sufficient molecular interactions with TRAX molecule. Thus, the fucose moiety of GHCer imposes complementary conformation of other sugar parts in GHCer conducive for interaction with TRAX protein, leading to the release of the sequestered PLCβ1 to promote angiogenesis. During biogenesis of GHCer, the terminal sugar fucose is added by fucosyltransferase (FUT) 1 and 2. Several reports suggest that *FUT1* and *FUT2* play important roles in angiogenesis [49, 50]. Our findings imply that GHCer is an important mediator by which *FUT1* and *FUT2* contribute to increased angiogenesis.

The impacts of fucose on the molecular interactions between GHCer and TRAX were also reflected in the high binding affinity. In general, binding affinities of carbohydrate ligands for lectins are usually quite low with Kd between 0.1–1 mM [51]. It has been suggested that multi-valent interactions are generated for carbohydrate ligands to attain biologically relevant affinity [51]. For example, increasing three or four terminal Gal residues on the complex-type N-glycans could raise approximately 10^5^-fold greater affinities than the monovalent galactoside for the binding of Gal/GalNAc-binding hepatic lectin to rabbit hepatocytes [51]. Intriguingly, GHCer, which possesses only one monovalent fucose at the terminus, conferred about 1000-fold higher affinity for TRAX than SSEA3Cer (Kd was 10^–8^ vs. 10^–5^ M). We also demonstrated that the concentration of secreted GHCer in culture medium of cancer cells was as high as 9.1 μM, far exceeding the Kd for GHCer binding to TRAX (40.9 nM). The actual concentration of GHCer in tumor microenvironment with tightly packed tumor clusters is likely to be even greater.

In this study, we have identified GHCer as a novel binding partner of intracellular TRAX protein at both cellular and molecular levels. Detailed binding studies between the recombinant proteins further verified the competition between GHCer and PLCβ1 for TRAX binding. In the Gαq/PLCβ/Ca^2+^ signaling pathway, PLCβ1 is distinctively different from other members of PLCs, because the former possesses a special C-terminal domain, where several binding partners will interact with [52]. It is also known that interaction of TRAX with this C-terminal domain of PLCβ1 in cytosol prevents the latter from association with Gα_q_ protein to incur Ca^2+^ influx [7]. In this study, we demonstrated the competition with PLCβ1 for binding to TRAX by either GHCer, or tumor-secreted EVs containing GHCer, resulting in the release of the TRAX-sequestered PLCβ1, leading to Ca^2+^ mobilization in endothelial cells and enhanced angiogenesis in tumor microenvironment. This viewpoint was further supported by our previous results which calcium influx induced by GHCer was abolished by preincubation of HUVECs with PLC inhibitor, U73122 [21]. Since EVs/GHCer-mediated angiogenesis is linked to TRAX/PLCβ1/Ca^2+^ signaling pathway, development of novel anti-cancer drugs that could disrupt the GHCer/TRAX interaction may provide a new strategy for the treatment of GHCer-positive cancer. Based on the prediction of molecular docking, PLCβ1 mainly interacted around the region where the TRAX accommodated the ceramide tail of GHCer [21]. Our current study revealed that the glycan part of GHCer formed H-bonding with key amino acid residues Q223 and Q219 on TRAX. These results suggest that if small chemical compounds could be found to fit into the unique glycan-binding site near Q223 and Q219 of TRAX, the binding of GHCer to TRAX could be blocked while the complex between TRAX and PLCβ1 would not be disrupted. Thus, structure-based design of small molecules that bind to the lower part of α3, α5, α6, and α7 helices of TRAX can be developed as novel agents for Globo H-targeted therapy to inhibit the angiogenesis in tumor microenvironments mediated by GHCer.

## Conclusions

This study deciphered the detailed molecular mechanism underlying GHCer-induced angiogenesis, which was mediated via transfer of GHCer from tumor derived EVs. The findings illustrated the important contribution of the terminal fucose moiety in GHCer to the glycan conformational change, which is essential for its interaction with TRAX, thereby releasing the TRAX-sequestered PLCβ1, leading to Ca^2+^ mobilization in endothelial cells and enhancement of angiogenesis.

## Supplementary Information

**Additional file 1. **Additional information for figures. This file contains additional information for Figures S1 to S4.

**Additional file 2.** Movie for GHCer. This file shows the MD simulation for GHCer in solution.

**Additional file 3.** Movie for TRAX-GHCer complex. This file shows the MD simulation for TRAX-GHCer complex.

**Additional file 4. **Movie for TRAX-SSEA3Cer complex. This file shows the MD simulation for TRAX-SSEA3Cer complex.

**Additional file 5.** Movie for GHCer binding to mutant TRAX. This file shows the MD simulation for GHCer binding to mutant TRAX.

## Data Availability

All data generated or analyzed during this study are included in this published article and its additional information files.

## Abbreviations

AP: Alkaline phosphatase
BSA: Bovine serum albumin
ELISA: Enzyme-linked immunosorbent assay
ER: Endoplasmic reticulum
EVs: Extracellular vesicles
FGF: Fibroblast growth factor
FRET: Fluorescence resonance energy transfer
FUT: Fucosyltransferase
GHCer: Globo H ceramide
GSLs: Glycosphingolipids
HUVECs: Human umbilical vein endothelial cells
IP_3_: Inositol-1,4,5-triphosphate
KDR: Kinase insert domain receptor
mAb: Monoclonal antibody
MD: Molecular dynamics
PBS: Phosphate buffered saline
PDX: Patient-derived xenograft
PIP_2_: Phosphatidylinositol-4,5-biphosphate
PLC: Phospholipase C
SSEA3Cer: Stage-specific embryonic antigen 3
TRAX: Translin-associated factor X
VEGF: Vascular endothelial growth factor
