# Supplementary material for: Conformational alteration in glycan induces phospholipase Cβ1 activation and angiogenesis
Source: J Biomed Sci. 2022 Dec 15;29:105. doi: 10.1186/s12929-022-00889-w (PMC9753400; doi:10.1186/s12929-022-00889-w)
Supplement: Supplementary file 1 — Additional file 1. Additional information for figures. This file contains additional information for Figures S1 to S4. [file 12929_2022_889_MOESM1_ESM.pdf]

Supporting Information for

Conformational Alteration in Glycan Induces Phospholipase Cβ1 Activation and Angiogenesis

Sheng-Hung Wang,[†1] Jing-Yan Cheng,[†1] Hsiu-Hui Tsai,[1] Tzu-Chi Lo[1], Jung-Tung Hung,[1] Chun-Cheng Lin,[2], Chien-Wei Lee,[1] Yi-Hsuan Ho,[1] Huan-Hsien Kuo,[1] Alice L. Yu,[1, 3] John Yu[1, 4, *]

[1] Institute of Stem Cell and Translational Cancer Research, Chang Gung Memorial Hospital at Linkou, and Chang Gung University, Taoyuan 333, Taiwan

[2] Department of chemistry, National Tsing Hua University, Hsinchu, Taiwan

[3] Department of Pediatrics, University of California in San Diego

[4] Institute of Cellular and Organismic Biology, Academia Sinica, Taipei, Taiwan

*Please address all correspondence to John Yu:

Dr. John Yu

Institute of Stem Cell and Translational Cancer Research

Chang Gung Memorial Hospital at Linkou, Taoyuan 333, Taiwan

TEL: 886-3-3281200 ext. 7803

E-mail: johnyu@gate.sinica.edu.tw

This Supporting Information includes:

Figures S1 to S4

Figures

Fig. S1

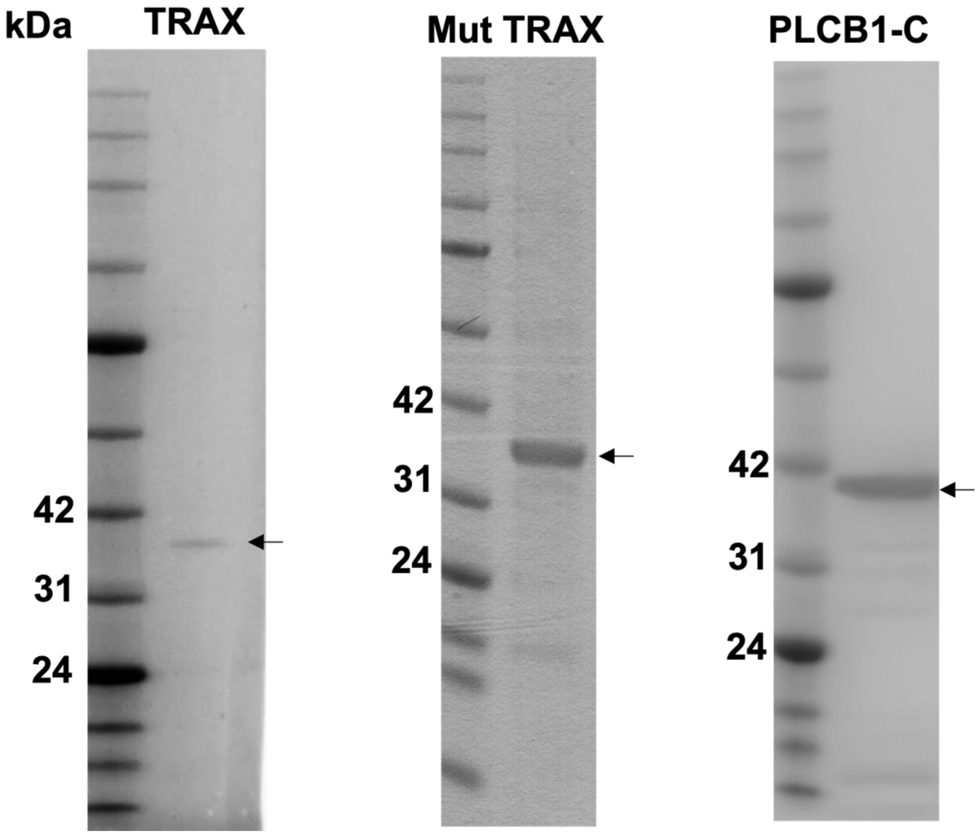

Fig. S1. The quality of purified recombinant TRAX protein (33 kDa, left panel), Mut TRAX (33 kDa, midddle panel), and PLCβ1-C (33 kDa, right panel) as analyzed by reducing gel stained with Coomassie Blue. The double mutations of Mut TRAX and the C-terminal domain of PLCβ1 (PLCβ1-C) were validated by sequencing analysis.

Fig. S2

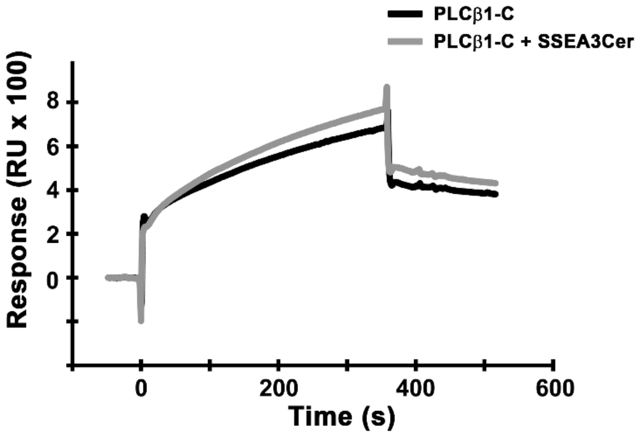

Fig. S2. Biacore analysis for competition between SSEA3Cer and PLCβ1-C for binding with TRAX. The binding of PLCβ1-C (333.3 nM) was not affected significantly by the presence of SSEA3Cer (10 μM), indicating that SSEA3Cer had no competitive effects on PLCβ1-C binding.

Fig. S3

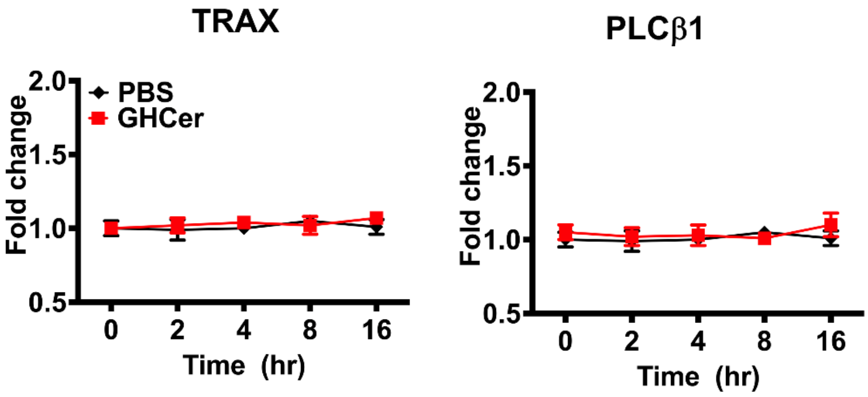

Fig. S3. mRNA expression levels of TRAX and PLCβ1 in GHCer-treated HUVECs. The mRNA expression levels of TRAX and PLCβ1 in GHCer-treated HUVECs were measured by real-time PCR. The TRAX and PLCβ1 mRNA remained unchanged during the time period examined (0-16 hr) after GHCer treatment.

Fig. S4

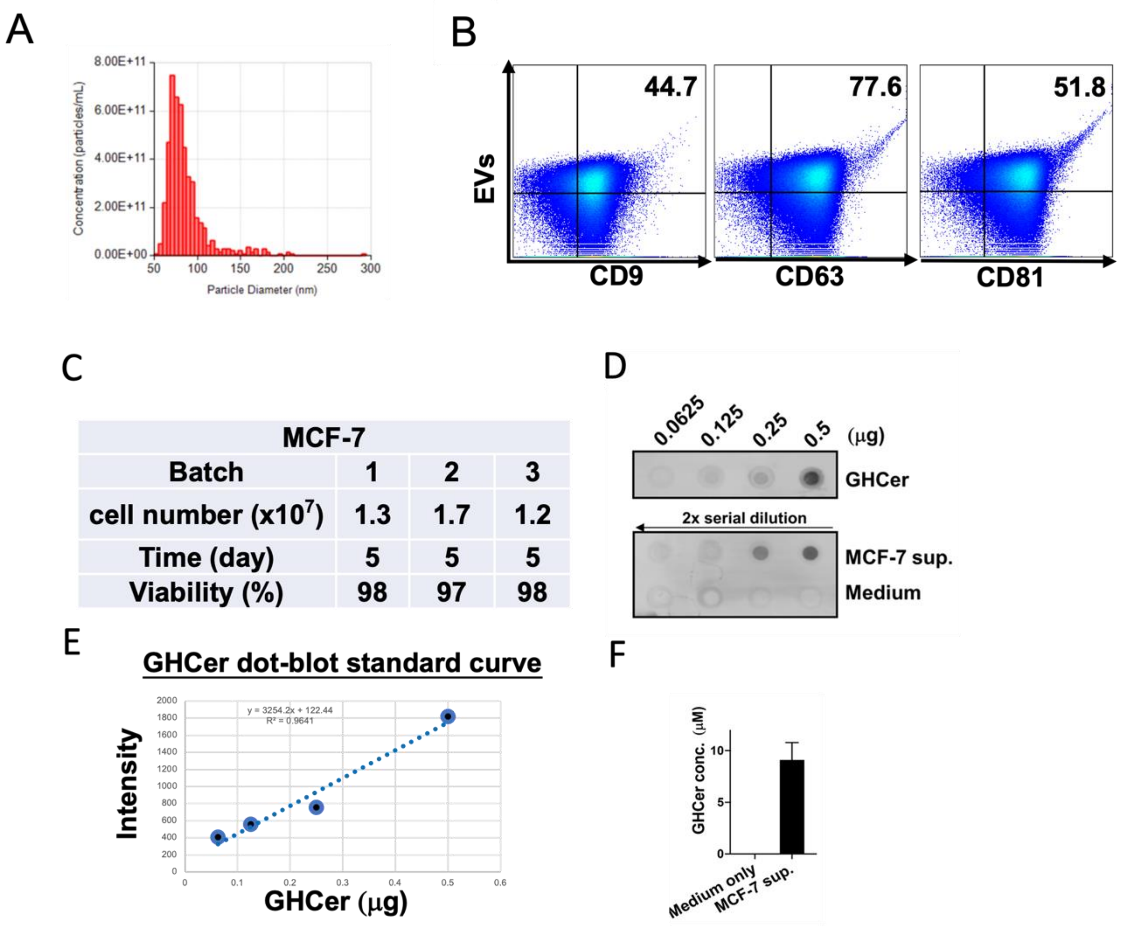

Fig. S4. Characterization of MCF7-secreted EV and GHCer concentration in conditioned medium of MCF-7. (A) The particle size distribution of EVs by Tunable Resistive Pulse Sensing. (B) NanoFlow analysis of exosomal markers (CD63, CD81, CD9). (C) Three batches of MCF-7 cells, after 5 days of culture, supernatant was centrifuged and filtered through 0.22 µm filters to remove cell debris. (D) Dot-blot analysis. Serial dilutions of the synthetic GHCer, the conditioned medium of the confluent MCF-7 cultures collected on day 5, and the medium alone, were probed with mAb VK9. Signals from GHCer standards were quantified with the ImageQuant software. The signal volume is the integration of

signal intensity over the area of the dot. (E) The linear regression curve yields an equation as presented. (F) Based on this equation, each sample signal in the linear range was converted to an absolute quantity of GHCer.
